# A Study on the Maximum Reliability of Multi-UAV Cooperation Relay Systems

**DOI:** 10.3390/s24092886

**Published:** 2024-04-30

**Authors:** Ning Ning, Suiping Zhou, Weimin Bao, Xiaoping Li

**Affiliations:** School of Aerospace Science and Technology, Xidian University, Xi’an 710126, China; spzhou@xidian.edu.cn (S.Z.); sast@xidian.edu.cn (W.B.); xpli@xidian.edu.cn (X.L.)

**Keywords:** UAV relaying platform, air-ground communication, line-of-sight, path loss, bit error rate

## Abstract

This paper studies the maximum reliability of multi-hop relay UAVs, in which UAVs provide wireless services for remote users as a coded cooperative relay without an end-to-end direct communication link. In this paper, the analytical expressions of the total power loss and total bit error rate are derived as reliability measures. First, based on the environmental statistical parameters, a LOS probability model is proposed. Then, the problem of minimizing the bit error rate of static and mobile UAVs is studied. The goal is to minimize the total bit error rate by jointly optimizing the height, elevation, power and path loss and introducing the maximum allowable path loss constraints, transmission power allocation constraints, and UAV height and elevation constraints. At the same time, the total path loss is minimized to achieve maximum ground communication coverage. However, the formulated joint optimization problem is nonconvex and generally difficult to solve. Therefore, we decomposed the problem into two subproblems and proposed an effective joint optimization iteration algorithm. Finally, the simulation results are given, and the analysis shows that the optimal height of different reliability measures is slightly different; thus, using the mobility of UAVs can improve the reliability of communication performance.

## 1. Introduction

Due to their strong mobility, flexible deployment on demand, low cost, line-of-sight (LoS) air-to-ground (AtG) link and high mobility, unmanned aerial vehicles (UAVs) have been widely used in civil and commercial applications in assisted wireless communication in recent years [1,2]. UAVs are the next generation of air platforms being deployed around the world, and there is a focus on developing smaller and lighter versions of existing systems. Therefore, light UAV base stations/relays have been conceived as revolutionary technologies for future wireless communication systems [2]. Air communication platforms built using light and small UAVs are a new means of constructing communication systems between existing ground communication systems and satellite communication systems. They are the best choice in emergency communication scenarios. They have the advantages of rapid deployment and flexible deployment and require less staffing.

UAV-enabled wireless communication platforms can provide greater wireless connections in areas where there is no communication infrastructure coverage or the communication infrastructure is damaged, and they can achieve higher LoS communication link capacity with ground terminals (GTs) [1,2,3,4]. Based on wireless relay technology, UAVs equipped with micro base stations and micro relays for cooperative communication, which extends the communication distance, further increases the low-altitude coverage advantage, and enhances the communication performance, can achieve a greater range of wireless communication coverage and higher communication link capacity and better communication performance [1]. Untethered UAVs generally rely on onboard batteries and solar energy collectors for power supply and are limited by current battery technology. The fact that tethered UAVs use cables to transmit electricity and signals solves the major problems of flight duration, safety, and reliability. China’s three major operators have conducted joint exercises and tests many times and have purchased tethered UAV aerial base stations for emergency communications.

At the same time, extensive research in academia has been dedicated to the use of UAVs in different types of air communication platforms, such as mobile base stations (MBSs) and mobile relays (MRs). In order to realize the potential of air communication platforms, allocating resources to UAV-assisted communication networks is crucial, which has also sparked research on the joint optimization of communication resource allocation [5,6,7,8,9]. The authors of [5] studied energy-efficient UAV communications and optimized resource allocation to maximize the energy efficiency of a UAV relay. The authors of [6] applied multiple UAVs to extend the duration of communications via UAV collaboration and optimized resource allocation to maximize end-to-end throughput. In [7], the authors considered a single UAV launched from a fixed initial location to a final location and optimized resource allocation to minimize the average outage probability of the system. In [8], the authors optimized resource allocation to maximize the service time duration of a UAV while simultaneously ensuring adequate coverage. In [9], the authors studied the use of a multi-hop UAV relaying network, and optimized resource allocation to maximize the total system throughput between two source nodes. Little of the existing research has focused on the effective deployment of UAVs as MRs, and the maximum reliability problem to be addressed by optimizing resource allocation is even less studied. However, the challenging fact concerns utilizing the mobility of UAVs, and the future research direction of UAV-assisted ground communications is in multi-UAV cooperative communications.

**Figure 1:**
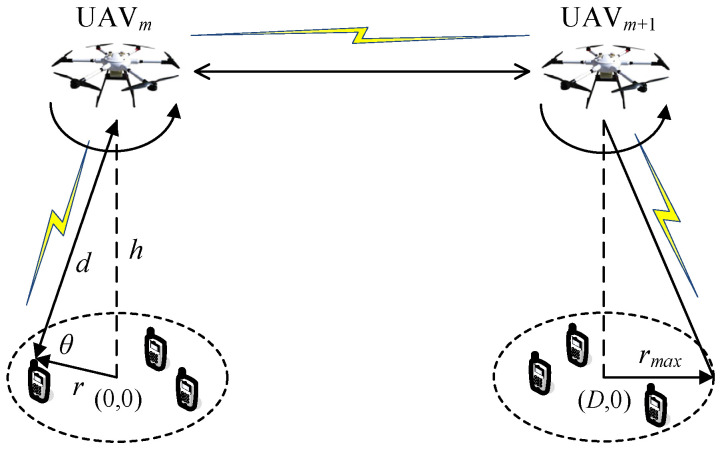
The considered UAV communication system.

In this paper, we consider a multi-source, multi-destination and multi-hop communication model in which the UAV acts as the coding cooperation (CC) relay, which can realize an air-to-air (AtA) relay to expand wireless coverage, as shown in Figure 1. This paper studies the problem of minimizing the BER of static and mobile UAVs, which is constrained by the maximum allowable path loss, the transmission power allocation, the UAV altitude and elevation, and minimizes the total path loss to achieve maximum communication coverage. However, the formulated optimization problem is nonconvex and generally difficult to solve. Firstly, two subproblems, namely, the height and elevation subproblem of given power and path loss, and the power and path loss subproblem of given height and elevation, are studied, which are processed by applying the gradient descent method and interior point method. Then, an effective joint optimization iteration algorithm is proposed, which can effectively update the optimization variables in each iteration and ensure the convergence of the algorithm. Finally, the effectiveness of the proposed algorithm is proved, which shows its superiority in the interrupted performance of mobile UAVs.

Notations: In this paper, scalars are denoted using italic letters, while vectors and matrices are, respectively, denoted using bold-face lower-case and upper-case letters. For vector a, $\left\|a\right\|$ represents its Euclidean norm, and $a^{T}$ denotes its transpose.

## 2. System Model and Problem Formulation

In order to study the UAV communication system, the radio propagation characteristics and LoS propagation characteristics of the UAV communication platform were studied first.

### 2.1. Modeling Line of Sight Probability

**Figure 2:**
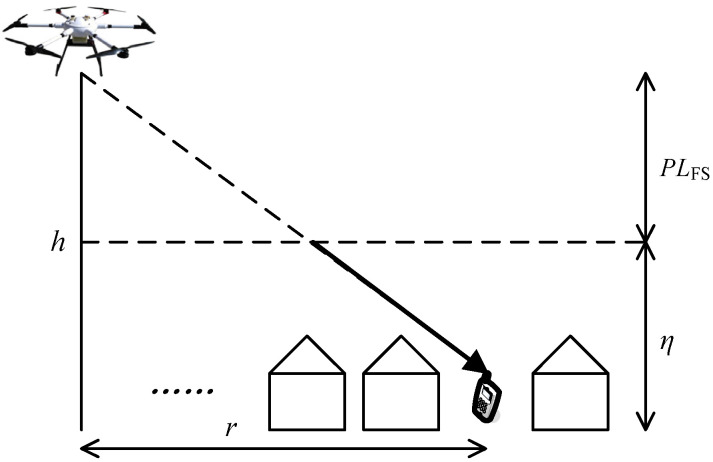
Radio propagation of UAV aerial base station platform.

UAV communication platforms are low-altitude platform (LAPs); that is, the flight area is within low altitude airspace. AtG communication occurs in accordance to two main propagation groups according to whether they adhere to struct LoS conditions, while the non-line-of-sight (NLoS) conditions correspond to receivers with no UAV platform LoS but still receiving coverage via strong reflections and diffractions [10,11]. The AtG communication channel from the UAV platform to the GTs is mainly dominated by LoS links. Radio signals emitted by the UAV platform base station propagate in free space, producing the free space path loss and reaching the ground building, incurring shadowing and scattering, which introduces an additional loss in the AtG link related to excessive path loss denoted by η [11,12], as shown in Figure 2. Accordingly, the resulting path loss of the AtG communication (expressed in dB) can be modeled as follows:

(1) $$PL_{\xi }=PL_{FS}+\eta_{\xi },$$

where $PL_{FS}$ represents the path loss of the free space between the UAV and ground users (GUs), and ξ refers to the propagation groups the propagation groups, which is classified according to the strict ground receiver LoS and NLoS conditions, while $\xi \in \left\{LoS,NLoS\right\}$. The maximum allowable path loss threshold was set to ${PL}_{max}$, which corresponds to a circular coverage with radius $r_{max}$. It can be noticed that the path loss between the UAV and any ground receiver within this circular coverage is less than or equal to ${PL}_{max}$. It is important to note that the value of ${PL}_{max}$ depends on the sensitivity of the receiver and the target communication’s quality of service. Assuming the use of isotropic transmitters and receiver antennas, the total path loss between the UAV platform and all ground receivers can be measured as the mean value of the path loss, as follows:

(2) $$PL=\sum_{\xi }{PL}_{\xi }P_{\xi },$$

where $P_{\xi }$ represents the probability of the occurrence of a certain propagation group ξ, while $\sum P_{\xi }\;=1$, ${PL}_{\xi }=20\log\left(\frac{4\pi fd}{c}\right)+\eta_{\xi }$, $d=\sqrt{h^{2}+r^{2}}$ is the link distance between the UAV LAP and GUs, h is the UAV LAP altitude, and r is the ground distance between the UAV platform and GUs.

In an urban environment, the probability of the geometric LoS between the ground transmitter at altitude $h_{TX}$ and the receiver at altitude $h_{RX}$ depends on three statistical parameters: α, β, and γ, which are related to the urban environment [10]. The probability $P_{LoS,n}$ that the height of an obtrusive building is smaller than height $h_{LoS,n}$ is given by

(3) $$P_{LoS,n}=1-exp\left(-\frac{h_{LoS,n}^{2}}{{2\gamma }^{2}}\right),$$

where $h_{LoS,n}$ is the height of a building that would obstruct the LoS in meters, while $h_{LoS,n}=h_{TX}-\frac{\left(n+\frac{1}{2}\right)\left(h_{TX}-h_{RX}\right)}{N_{n}}$, $N_{n}$ is the number of buildings crossed by the LoS, while $N_{n}=floor\left(r\sqrt{\alpha \beta }\right)$. Parameter α is the ratio of land area covered by buildings to the total land area (dimensionless). Parameter β is the mean number of buildings per unit area (buildings/km^2^), r=h/tan(θ), while θ is the elevation angle. Parameter γ is a variable used to determine the height distribution of obtrusive buildings, which is defined by the Rayleigh probability density function (PDF) of the height $h_{LoS,n}$, namely $f\left(x\right)=\frac{x}{\gamma^{2}}exp\left(-\frac{x^{2}}{{2\gamma }^{2}}\right)$.

The probability $P_{LoS}$ of having LoS rays at position $r\;\leq \;r_{max}$ for GUs is given by

(4) $$P_{LoS}=\prod_{0}^{N_{n}-1}P_{LoS,n},$$

**Figure 3:**
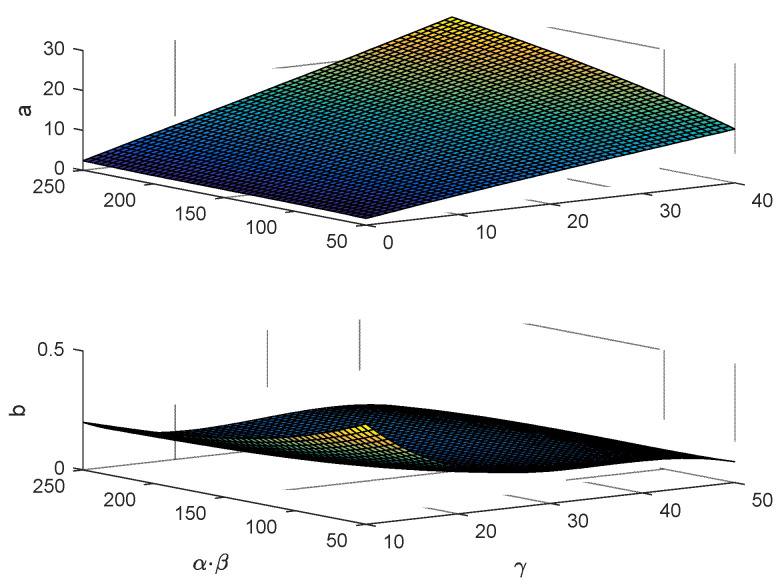
Three-dimensional fitting of the S-curve parameters and the environmental parameters.

Equation (4) can be considered a continuous function of the elevation angle and the three environment parameters and can be used for any $h_{TX}$ and $h_{RX}$ heights. In the case of the UAV air base station platform, we can disregard $h_{RX}$ since it is much lower than the altitude of the UAV platform. However, the series in Equation (4) cannot be further reduced; therefore, it is necessary to simplify the calculation of LoS probability. Note that the trend of LoS probability can be closely approximated to a modified Sigmoid (S-shape) function with the following form:

(5) $$P_{LoS}\left(\theta \right)=\frac{1}{1+aexp\left(-b\left[\theta -a\right]\right)},$$

where $a=\sum_{j=0}^{3}\sum_{i=0}^{3-j}A_{ij}{\left(\alpha \beta \right)}^{i}\gamma^{j}$ and $b=\sum_{j=0}^{3}\sum_{i=0}^{3-j}B_{ij}{\left(\alpha \beta \right)}^{i}\gamma^{j}$ are the fitting parameters, while $A_{ij}$ and $B_{ij}$ are the polynomial coefficients. In order to link the fitting parameters with environmental parameters, surface fitting can be carried out using two variables (α×β) and (γ), as shown in Figure 3.

Substituting Equation (5) into Equation (2), we can obtain the following:

(6) $$PL=\frac{\eta_{LoS}-\eta_{NLoS}}{1+aexp\left(-b\left[\theta -a\right]\right)}+20\log\left(\frac{4\pi fd}{c}\right)+\eta_{NLoS},$$

where f is the carrier frequency and c is the speed of light.

### 2.2. Modeling UAV Communication System

In this paper, we study a multi-hop communication system relating to a tethered multi-rotor UAV low-altitude emergency communication platform, where the UAV acts as the air MBSs and MRs and *M* ≥ 1 UAVs are deployed in a given geographical area within a given limited duration *T* to collaboratively serve a group of *K* > 1 GUs at altitude *h*. Each UAV is equipped with a micro base station to provide communication coverage for GUs within the circle of the maximum coverage radius $r_{max}$ in Figure 1, where the radius of GUs in polar coordinates is $r_{G}$ and the angle is $\alpha_{G}$. If the UAV is static, it hovers over the center of the circular coverage area; if the UAV is mobile, it circles over the circular coverage area with radius $r_{U}$ and angle $\alpha_{U}$, and flies in one direction at its maximum speed V. This multi-UAV collaborative communication system equipped with dual antennas adopts a 2.4 GHz microwave communication frequency band and space-time block coding (STBC). Assuming the use of M-base modulation, each *m* bits first in the binary information bit stream transmitted by the information source are divided into a group, and two consecutive sets of bits are modulated and mapped; that is, each set of bits is mapped to a modulating symbol on the constellation diagram. Therefore, we obtain two modulation signals, $x_{1}$ and $x_{2}$, and the method used for encoding is as follows:

(7) $$X=\left[\begin{array}{cc} x_{1} & x_{2}\\ -x_{2}^{*} & x_{1}^{*} \end{array}\right],$$

where X is the encoding matrix and $x_{1}^{*}$ represents the complex conjugate of $x_{1}$.

UAVs and GUs constitute a multi-source multi-destination multi-hop relay, as shown in Figure 1, where it is assumed that GU*_i_* is the source node or the destination node, GU*_j_* acts as the destination node or source node according to the communication direction, while UAV*_m_* and UAV*_m_*_+1_ act as relays in both directions, and the center of the circular coverage area of UAV*_m_* is the origin of the coordinate system. The coordinates for the GU*_i_* and GU*_j_* are $\left(r_{i}\cos\alpha_{i},r_{i}\sin\alpha_{i},\;0\right)$ and $(D+r_{j}\cos\alpha_{j},\;r_{j}\sin\alpha_{j},\;0),respectively$, where *D* denoting the distance between the centers of the coverage areas of UAV*_m_* and UAV*_m_*_+1_. For a static UAV, the coordinates for the UAV*_m_* and UAV*_m_*_+1_ are $\left(0,\;0,h_{m}\right)$ and $(D,\;0,\;h_{m+1}),respectively$. For a mobile UAV, the coordinates for the UAV*_m_* and UAV*_m_*_+1_ are $\left(r_{m}\cos\alpha_{m},r_{m}\sin\alpha_{m},\;h_{m}\right)$ and $(D+r_{m+1}\cos\alpha_{m+1},r_{m+1}\sin\alpha_{m+1},\;h_{m+1}),respectively$, where $\alpha_{m}$ and $\alpha_{m+1}$ is the angle between the UAV and the X axis. Using CC, UAV*_m_* demodulates and decodes the information sent by GU*_i_*, and then encodes the channel and forwards it to UAV*_m_*_+1_. UAV*_m_*_+1_ demodulates and decodes the received information, then encodes the channel and then forwards it to GU*_j_*. The communication link’s signal-to-noise ratio (SNR) is as follows:

(8) $$\gamma_{n}=\frac{P_{TX}{\left\|g_{n}\right\|}^{2}}{\sigma^{2}Q_{n}},$$

where $g_{n}$ is the channel fading coefficient, $\sigma^{2}$ is the noise variance, and the power loss $Q_{n}$ satisfies ${PL}_{n}=10\log_{10}Q_{n}$, while $n\in \left\{1,2,3\right\}$, *n* = 1, *n* = 2, and *n* = 3 correspond to the uplink, AtA, and downlink conditions, respectively. Assuming that the AtG communication links are Nakagami-*k* fading channels, k∈[0.5,1), the PDF of $\left\|g_{n}\right\|$ is as follows:

(9) $$f_{\left\|g_{n}\right\|}\left(x\right)=\frac{2{k_{n}}^{k_{n}}x^{2k_{n}-1}}{\Gamma \left(k_{n}\right){\Omega_{n}}^{k_{n}}}e^{-\frac{k_{n}x^{2}}{\Omega_{n}}},$$

where x≥0, Γ(·) is the Gamma function, $k_{n}$ is the shape parameter of Nakagami fading and indicates the severity of fading, and $\Omega_{n}=E\left\{{\left|g_{n}\right|}^{2}\right\}$ is the average fading powers. Using (8) and (9), the PDFs of $\gamma_{n}$ obeying the gamma distribution are as follows:

(10) $$f\left(x|y_{n},z_{n}\right)=\frac{x^{y_{n}-1}e^{-\frac{x}{z_{n}}}}{{z_{n}}^{y_{n}}\Gamma \left(y_{n}\right)},$$

where $y_{n}$ is shape parameter and $z_{n}$ is scale parameter. The cumulative distribution function (CDF) of $\gamma_{n}$ can be derived as follows:

(11) $$F_{\gamma_{n}}\left(x\right)=\Gamma \left(\frac{x}{z_{n}},y_{n}\right),$$

where Γ(·,·) is the incomplete Gamma function. By averaging the instantaneous BER of the joint PDF of link SNR $\gamma_{1}$, $\gamma_{2}$, and $\gamma_{3}$, the total BER can be obtained as follows:

(12) $$P_{e}=H\left(z_{1},\;y_{1}\right)+H\left(z_{2},\;y_{2}\right)+H\left(z_{3},\;y_{3}\right)-2H\left(z_{1},\;y_{1}\right)H\left(z_{2},\;y_{2}\right)-2H\left(z_{1},\;y_{1}\right)H\left(z_{3},\;y_{3}\right)-2H\left(z_{2},\;y_{2}\right)H\left(z_{3},\;y_{3}\right)$$

where Hzn, yn=12−12zn1+zn∑N=0yn−12NN41+z1N, while $y_{n}=k_{n}$ and $z_{n}=\frac{\Omega_{n}}{2\sigma^{2}{k_{n}Q}_{n}}$.

### 2.3. Optimization Problem Formulation

In UVA-assisted multi-hop communication systems without reliable direct communication links, there is no need for a reliable direct communication link. In order to obtain the maximum system reliability, the goal is to minimize the total BER by jointly optimizing the height **H**, elevation θ, transmission power **P**, and path loss **PL** and subject these to the maximum allowable path loss constraints, transmission power allocation constraints, and UAV height and elevation constraints. At the same time, the coverage radius should be maximized and the total path loss should be minimized.

(13) $$\underset{H,\theta ,P,PL}{min}\;P_{e},$$

(13a) $$s.t.\;\;0\leq P_{G}\leq P_{max},\;0\leq P_{U}\leq P_{max},$$

(13b) $${PL}_{n}\leq {PL}_{max},\;\forall n\in \left\{1,2,3\right\},$$

(13c) $$r=\frac{h}{tan\left(\theta \right)}\leq {\left.r_{max}\right|}_{PL_{max}},$$

(13d) $$\theta_{min}\leq \theta \leq \theta_{max},\;H_{min}\leq H\leq H_{max}$$

where (13a) are power constraints, (13b) are maximum allowable path loss constraint, and (13c) and (13d) are UAV height and elevation constraints.

## 3. Joint Optimization

Equation (13) shows that the joint optimization problem (13) is nonconvex with respect to (**H**, **θ**, **P**, and **PL**). In order to deal with it more effectively, a joint optimization algorithm is proposed. Algorithm 1 illustrates the algorithm. This problem is solved by alternating minimization, in which the joint optimization problem (13) is decoupled into two subproblems, namely, the height and elevation subproblem with given power and path loss, and the power and path loss subproblem with given height and elevation. It is solved by using the gradient descent method and interior point method. In each iteration, the optimization variables are updated effectively by giving the last iteration, and the convergence of the algorithm is guaranteed.

### 3.1. Height and Elevation Optimization Subproblem

Given the power variables $P_{G}$ and $P_{U}$, and the path loss variable PL, the Equation (13) can be expressed as follows:

(14) $$\underset{H,\theta }{min}\;\;P_{e},$$

(14a) $$s.t.\;\;r\;=\;h/tan(\theta )\leq {\left.r_{max}\right|}_{PL_{max}},$$

(14b) $$\theta_{min}\leq \theta \leq \theta_{max},\;H_{min}\leq H\leq H_{max}$$

This is a convex optimization problem, which can be solved by using standard convex optimization techniques. In order to achieve the local minimum BER, the descent direction is $-\nabla P_{e}$, and the step size of the gradient descent process is set to υ until the stop condition is met. A minimum BER threshold is also set δ, $\delta \to 0^{-}$. When $-\nabla P_{e}^{t}\geq \delta$, the minimum BER is considered to have been reached. There are optimal height $h^{★}$ and elevation $\theta^{★}$, and the corresponding BER is minimized, which can be obtained by searching $\left[h_{min},h_{max}\right]$ and $\left[\theta_{min},\theta_{max}\right]$ and satisfying $\frac{\partial P_{e}}{\partial h}=0$ and $\frac{\partial P_{e}}{\partial \theta }=0$.

### 3.2. Power and Path Loss Optimization Subproblem

Given height variable h and elevation variable θ, Equation (13) can be expressed as follows:

(15) $$\underset{P,PL}{min}\;\;P_{e},$$

(15a) $$s.t.\;\;0\leq P_{G}\leq P_{max},\;0\leq P_{U}\leq P_{max},$$

(15b) $${PL}_{n}\leq {PL}_{max},\;\forall n\in \left\{1,2,3\right\},$$

The BER function in Equation (15) is convex with respect to $P_{G}$ and $P_{U}$, which can be solved using standard convex optimization techniques. Therefore, the optimal power allocation is realized.

### 3.3. Joint Optimization Problem

As shown in Algorithm 1, the key idea of the proposed algorithm is to alternately optimize the height and elevation subproblem and the power and path loss subproblem. In each iteration, the main complexity of the proposed algorithm lies in steps 4 and 6, which is needed to solve a series of convex problems. In iteration l, let $S^{l}=\sum_{t=1}^{T}P_{e}^{t}$, when $S^{l}-S^{l-1}\leq \epsilon$, the joint optimization algorithm converges, where ϵ is a predefined error tolerance threshold.
**Algorithm 1:** The joint optimization algorithm1: Initialize l=0
$,\;S^{0}=0$
$,\;P_{G}^{t}=P_{U}^{t}=P_{max}$
, ∀t=1,…,T
2: Repeat 3:     l=l+1; 4:     For t=1:T 5:           solve the height and elevation subproblem (14). 6:     For t=1:T
7:           solve the power and path loss subproblem (15).8: Until $S^{l}-S^{l-1}\leq \epsilon$


## 4. Simulations and Discussions

In this section, simulations and analysis are conducted. Set time slot *T* = 1000, and the maximum moving distance of UAV in each time slot is υ = 0.1 m, V = 10 m/s, $\eta_{LOS}\in$ [0.1, 1.6] dB, $\eta_{NLOS}\in$ [21, 23] dB, α∈ [0.1, 0.3], β∈ [500, 750], γ∈ [8, 15], a∈ [0.1139, 0.4290], b∈ [4.8800, 12.0810], Ω = 25 mW, $\sigma^{2}$ = −96 dBm, $\gamma_{th}$ = 0 dB, ϵ = −10^−2^, $P_{max}\;$ = 26 dBm $r_{max}\;$ = 4000 m, ${PL}_{max}\;$ = 113 dB, and δ = −10^−2^.

**Figure 4:**
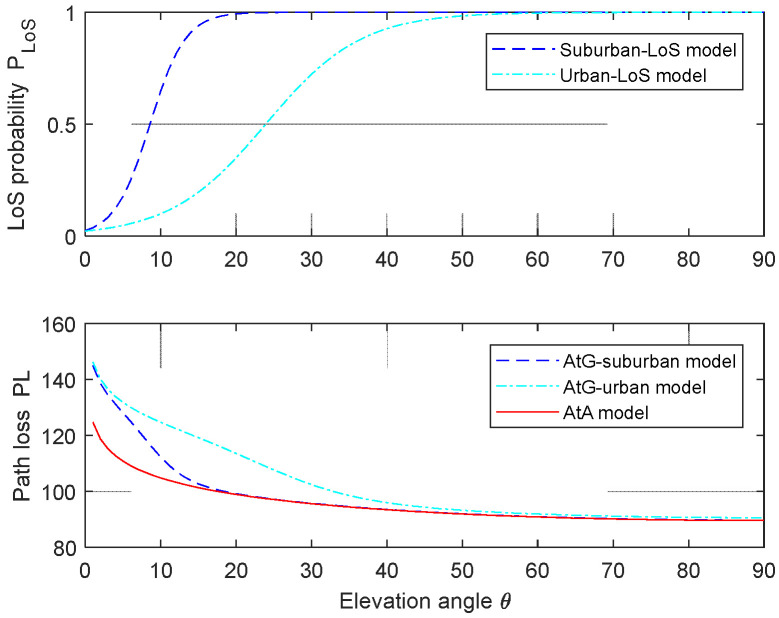
LoS probability and path loss for different environments model.

Figure 4 shows LoS probability and path loss under different environmental models. For the suburban and urban environment models, the LoS probability of the suburban environment increases more rapidly than that of the urban environment and then tends toward 1 with t increasing elevation angle, while the path loss of the suburban environment model decreases more rapidly than that of the urban environment model and then tends toward stability in the AtG model, and the path loss attenuation of the AtG model is faster than that of the AtG model. That is to say, AtA path loss is lower than AtG path loss, and the AtG path loss in suburban environments is lower than that in urban environments. When $\theta^{★}$, the corresponding path loss is minimized. Within the maximum coverage radius, the closer the ground user is to the geometric center of the ground, the smaller the radius, and the lower the path loss.

**Figure 5:**
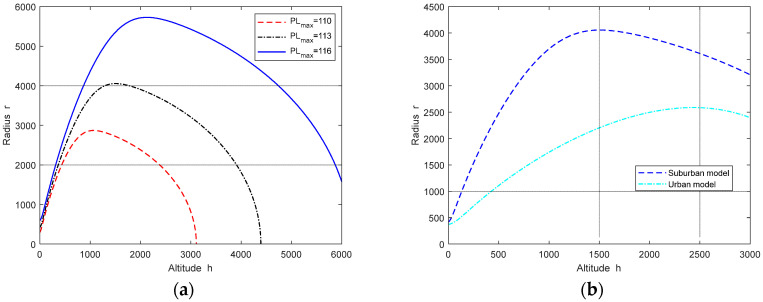
The effect of different $PL_{max}$ values and environments models on $h^{★}$: (**a**) radius vs. altitude curve for different $PL_{max}$ values; (**b**) radius vs. altitude curve for different environment models.

Figure 5 shows the effect of different $PL_{max}$ values and environment models on $h^{★}$. Figure 5a shows the change in unit radius relative to UAV height in the case of different $PL_{max}$ values. The point on the radius–height curve in the figure that changes its direction; specifically, the top of the curve is the optimal height solution: $h^{★}$. It can be seen from Figure 5a that the angle between the horizontal axis and the line generated by connecting the top of each radius–height curve to the origin represents the optimal elevation $\theta^{★}$ which always meets the constant ratio $\frac{h^{★}}{r}$. The larger the ${PL}_{max}$ value, the larger the $h^{★}$ value. Due to the main consideration of low-altitude airspace in this work, the LAP will be subject to physically constraints when reaching the optimal altitude. Therefore, there is $h^{★}$, and the corresponding path loss is at its minimum. Figure 5b shows the effect of suburban and urban environment models on $h^{★}$. It can be seen that $h^{★}$ is a function of the maximum allowable path loss and environmental statistical parameters, which largely depend on the environmental conditions in specific regions.

**Figure 6:**
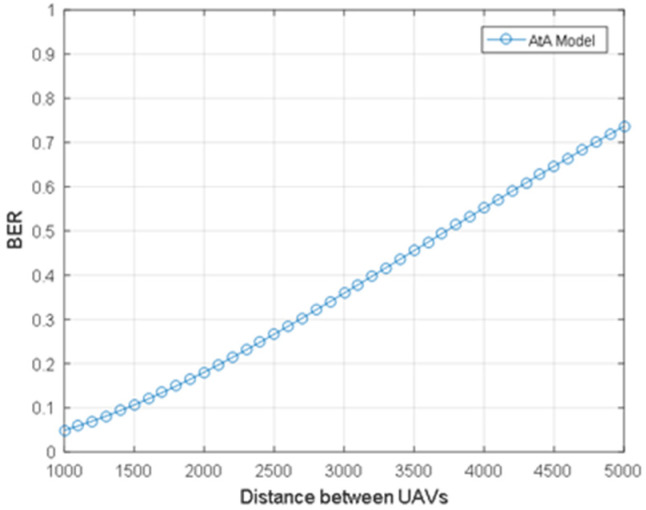
The BER for the AtA communication model.

Figure 6 describes the relationship between the distance between UAVs and the BER of AtA communication links. When the distance between UAVs decreases, the BER decreases. That is to say, the shorter distance leads to lower outage because when the distance decreases, AtA communication link loss decreases.

**Figure 7:**
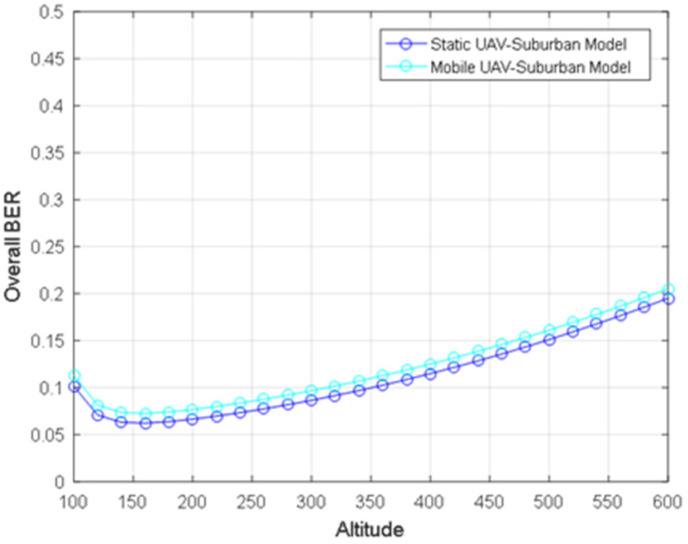
The downlink BER of static and mobile UAVs for a suburban environment.

Figure 7 describes the BER of the downlink communication links of static and mobile UAVs under a suburban environment model. When the UAV height increases, the BER first decreases to the minimum value and then increases slowly. The AtG communication link loss decreases to the minimum value and then increases slowly with the increase in UAV height. The path loss of the AtA communication link increases with the increase in UAV height, and its change is very small, with the path loss difference within 0.1 dB. It can be seen from Figure 7 that the BER of a mobile UAV is slightly lower than that of a static UAV. The bottom of the curve is $h^{★}$, corresponding to the minimum BER.

**Figure 8:**
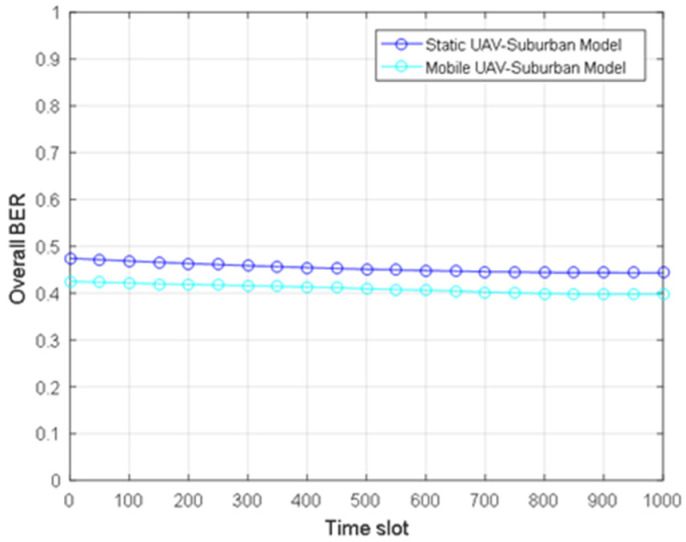
Time slot vs. overall BER of the static and mobile UAV for a suburban environment.

Figure 8 describes the total BER of static and mobile UAVs under a suburban environment model. Through the proposed joint optimization algorithm, the total BER decreases by about 5% with time, and the minimum BER is obtained, which tends to be stable at the minimum level. The results show that the total BER increases monotonously with the increasing distance between GUs, and the total BER of a mobile UAV is slightly lower than that of a static UAV, while the difference between the minimum BER of a static UAV and that of a mobile UAV is about 8%. The results also show that using the mobility of the UAV can improve communication performance, and the performance of a mobile UAV is better than that of a static UAV. In addition, it can be seen from Figure 4, Figure 7 and Figure 8 that the optimal height of different reliability measures is different; namely, the optimal height to minimize the total power loss is very different from the optimal height to minimize the total BER, and the optimal height to minimize the downlink BER and the total BER are also different.

## 5. Conclusions

This paper studied the problem of maximizing the reliability of static and mobile UAVs in a multi-source, multi-destination, and multi-hop communication model, in which the UAV provides wireless services for remote users without direct communication links on the ground as a CC relay. The goal was to minimize the total BER by jointly optimizing the variable and, at the same time, the total path loss was minimized and the ground communication coverage was maximized. The simulation results validated the superiority of the proposed joint optimization iteration algorithm and showed that the optimal height of different reliability measures is slightly different, and when using the mobility of UAVs, the outage performance of the mobile UAV is better than that of the static UAV and can improve the reliability of communication performance.

## Data Availability

The data presented in this study are available on request from the corresponding author.
